# What Keeps the Family Caregiver Motivated to Care for Their Dying Relative at Home? A Brief Report of a Qualitative Interview Study

**DOI:** 10.1089/pmr.2024.0009

**Published:** 2024-05-15

**Authors:** Julia Strupp, Alina Kasdorf, Jonas Karneboge, Raymond Voltz

**Affiliations:** ^1^ Department of Palliative Medicine, Faculty of Medicine and University Hospital, University of Cologne, Cologne, Germany.; ^2^ Department of Psychology, Psychological Aging Research (PAR), Faculty V: School of Life Sciences, University of Siegen, Siegen, Germany.; ^3^ Faculty of Medicine and University Hospital, Center for Integrated Oncology Aachen Bonn Cologne Duesseldorf (CIO ABCD), University Hospital Cologne, Cologne, Germany.; ^4^ Faculty of Medicine and University Hospital, Center for Health Services Research, University of Cologne, Cologne, Germany.

**Keywords:** caregiver, qualitative study, dying at home, home care, end of life

## Abstract

**Background:**

Dying at home poses many challenges for family carers and is particularly distressing for those with limited social support. In addition to financial hardship, this perceived burden may be a deciding factor in providing care at home.

**Aims:**

To explore what motivates people to provide care at home until death.

**Methods:**

Qualitative interviews with 43 family carers of deceased patients about factors enabling death at home. Interviews were audio-recorded, transcribed verbatim, and analyzed using content analysis.

**Results:**

Participants who rated their end-of-life experience positively reported that they particularly benefited from encouraging feedback and gratitude from their dying loved ones, as well as appraisal support. It takes courage to care for someone at home and to feel responsible for them. These themes made the participants’ home care efforts meaningful, gave them confidence in what they were doing and helped maintain their motivation to care.

**Conclusion:**

Encouraging feedback and appraisal support are both minimally invasive techniques with maximum impact for continuing care at home.

## Introduction

Research has shown that a key factor in enabling people to die at home is the availability and willingness of family members to take on the caregiving role for a person who wishes to die at home.^1^ Particularly the provision of home care in the evenings and at night is often limited and makes the involvement and commitment of family members necessary.^2^ But what keeps the carer motivated to provide and continue care at home?

According to Voltz et al., the majority of terminally ill patients (68%) prefer to die at home, often without life-sustaining treatments, but only a few (28%) succeed in doing so.^3^ Home-based care at the end of life has been shown to be a major challenge for some caregivers, who may feel homebound, isolated, stressed, and sleep-deprived.^1^ A major reason why patients are admitted to hospital when they want to die at home is a breakdown in family caregiving.^4,5^ According to Pearlin et al.,^6^ the conditions, experiences, responses, and resources that make up caregiver burden are thought to vary in their impact on the health and behavior of caregivers. Their model describes four important domains that lead to caregiver strain: first is the history and setting of the stress (important characteristics of the caregiver, nature of the dyadic relationship, duration of caregiving, level of support, and influence of other life events on coping capacity). The primary burden of the illness includes the patient’s cognitive capacity, the level of support and supervision provided, and any behavioral or psychiatric symptoms. Secondary role pressures (work, finances, family dynamics, relationships, and social life outside the caring role) are covered in the third section. The fourth section examines the caregiver’s intrapsychic stressors (personality, level of anxiety, and depression). A study by Pertl et al.^7^ reported that >80% of carers did not have a choice in taking on the caring role and that those who reported a greater degree of choice were more likely to still be providing care at home.

Little is known about how family members in Germany experience caring for a relative with a serious illness at home until death and what keeps them motivated to continue caring at home. Exploring their perspectives may help to better support them in providing optimal care and reduce caregiver burden, i.e., reduce mental and physical distress.

This qualitative study aimed to provide insight into conditions that made dying at home possible and to describe family caregivers’ experiences of providing care at home.

## Materials and Methods

This brief report is part of a mixed-methods research project “Dying at home”. The study included interviews conducted by two researchers experienced in qualitative methods (A.K.: research associate with a doctorate; J.S.: project leader and senior researcher with two doctorates) and a study nurse. They were unknown to all but one of the study participants at the time of data collection. Interviews were conducted online via Zoom, by telephone, or face to face. One informant provided written notes in lieu of an interview. Carers were included if they were adults with current or past (in the past three years) experience of caring at home for someone with a life-threatening illness (e.g., spouse, friend). Demographic data were collected and participants were asked whether they had positive view of their experience of caregiving at the end of life and what contributed to this experience. The study also focused on support options for patients and their carers, and when and where other services were needed.

Detailed eligibility criteria and study procedures have been described elsewhere.^8^ All participants were recruited via a newspaper article and flyers posted by the study collaborators. All informant groups were included in the study if they were over 18 years of age and had given written informed consent to participate in the study (hereafter referred to as “informants”).

Data collection was conducted until an adequate level of information power was achieved^9^ and continued between December 2021 and April 2022. Information power indicates that the more information the sample has that is relevant to the study at hand, the smaller the number of participants needed.^9^ The size of a sample with sufficient power depends, for example, on the aim of the study. As our study objective was rather broad, we initially aimed to purposively recruit 40 participants, also for ensuring heterogeneity. Throughout the process and supported by the preliminary analysis, the assessment of information power was repeated within the researchers (J.S., A.K., and J.K.).

We received a total of 120 expressions of interest in our study, 117 of whom were either current carers or bereaved carers, of whom interviews were conducted with 43 informants. The others either did not meet the inclusion criteria and were not included in the analysis, or withdrew their consent to participate and were not included in the dataset. All potential participants received invitations and an information sheet by e-mail or letter to encourage participation. During the semi-structured interviews, participants were asked about their experiences of caring for their loved one with a terminal illness at home. Carers were asked what support they had received, or would have liked to have received, while caring for their relative. They were also asked about positive and negative experiences with health care professionals during the care period. We also explored which supportive and encouraging responses had been perceived as particularly rewarding and whether this could be linked to the overall evaluation of the caring period. The transcripts were analyzed using qualitative content analysis. We analyzed the data in terms of factors that keep carers motivated and encouraged to continue caring at home. The qualitative data were audio-recorded, transcribed verbatim, and then coded using MAXQDA^©^. All locations, names, and identifiable information were anonymized during transcription and given a unique code. Ethics approval was granted by the Ethics Commission of the Faculty of Medicine of Cologne University on November 2021 (#21–1466).

## Results

The sample consisted of 43 family caregivers with a median age of 60 years; 72.1 percent were spouses or sons/daughters (39.5%/32.6%). Most (62.2%) had lived together with their ill family member. The desired place of death—at home—was known for 29 patients (64.5%). Of these 29, 24 died at home (82.8%).

A theme that emerged throughout the interviews was what is known as “appraisal support,”, which refers to the provision of feedback on performance or personal qualities.

*“Well, as I said, these various professional offers from outside. Of course, friends have also been incredibly helpful to me personally, encouraging me, supporting me mentally, so to speak.” (KD_1S41)*

*“I would have liked to have had a mum who would have taken me by the hand (crying). A couple of very good friends of mine took over that to a large extent, who also kept saying ‘You’re doing it right, that’s fine. Do it the way that's right for you.” (KD_1S41)*

*“(…) that she showed me other ways of thinking and said ‘Mrs., you’re doing it well, like this’-so she always gave me good encouragement. I always thought that was great, too, saying ‘You’re doing it just right.’ Yes, and that helped me a lot, it helped ME a lot, yes.” (KD_1S79)*

*“And in the end I said, it’s okay, she’s encouraging me now. (…) she said, ‘I think it’s so great how you did it.’ She always encouraged me in what I had done. And that’s also really important, that you have someone who believes in you and then also says that, not only believes, but also makes it known. And yes, that’s what I wish for everyone, that you have people like that who simply give you strength.” (KD_1S12)*

“Having the courage to care at home” was another factor that was mentioned as a reason for continuing care at home:

*“(…) well, I’m not brave as such, but I was made brave by my sister saying ‘You’re coming to me now’. (laughs) (…) that you decide for yourself individually what you think a dying person needs. Whether what I did was right, I don’t know, nobody knows. But it helped ME not just to sit there, but to actively do something for them, by reading to them, by lighting candles, (…) yes.” (KD_117)*

*“And I- I really wish for a lot of people that they dare to do what I just- just dared to do, right?” (KD_1S10)*

*“(…) that they are brave to take this step (…), (…) I think it’s important to be there for each other (KD_1S69).”*

“Feedback and gratitude from their dying relatives” were mentioned as well:

*What’s going well? Well, I’m used to it, my mum is very cooperative and — and grateful. And she’s also happy that she’s so well — she’s basically well looked after. And she says that and she’s happy about it. And she thanks me for that. And that makes it easier, of course, than if she was sort of prickly now. (Interview_KD_1S88: 17)*

Another theme mentioned was to “take the lead in care”:

*“Basically, I did everything. (KD_1S6)*

*“Also in the family I am the only one who is responsible for it. (…) And in the end everything is up to me. And I have to make decisions and know everything, be able to do everything.” (KD_1S4)*

Taking the lead, however, tends “to put you last.”

*“And not everyone can just give in like that, so, yes, I just gave up on myself at that time. It’s just that he came first and my needs were relatively secondary. And that was okay for the time. I would have done it longer, of course. But you have to be able to do that.” (KD_1S79)*

## Discussion

We explored the experiences of people caring for someone who had died or wanted to die at home and looked at themes contributing to family caregivers’ motivation to care for their dying loved ones at home. Our results show that participants who had a positive view of their experience of caregiving at the end of life reported that they had particularly benefited from receiving affirming feedback from their loved ones and, in particular, from health care professionals, and that this had proved meaningful to them. Our findings suggest that caregivers may benefit from positive and motivating interaction that gives them confidence in their actions and can help to maintain motivation to care.

Overall, the caregivers’ narratives support the idea that encouragement and self-belief are the most important factors in continuing to care for someone at home and enabling them to die at home. Caregivers reported that their focus was on their seriously ill relative, with themselves coming last. According to a study by Martín et al.,^10^ family members often felt abandoned while caring for a sick family member and neglected.

In agreement with the stress process model by Pearlin et al.,^6^ intrapsychic strains occur when the caregiver’s self-concept is diminished by the chronicity of caregiving. Two of these intrapsychic stressors specific to caregivers have been defined as (1) “loss of self,” in which the caregiver experiences a loss of identity and sense of personhood as they become involved with the patient, and (2) “perceived low competence,” in which the caregiver does not see the value and skill of the care they are providing, leading to a sense of helplessness. These stressors may be influenced by positive appraisal and feedback. Future studies should use these factors to analyze the effectiveness of encouragement and positive feedback in enabling care at home and thus, if preferred, death at home.

## Limitations

A limitation of the study is that it is unclear whether the different methods of conducting the interviews (virtual/face to face) had an impact on the results. Although the retrospective nature of the study may be seen as a drawback, it does allow for reflection by the carers surveyed, which may provide a more nuanced assessment of the advantages and disadvantages of home care until death.

## Conclusions

In order to maintain motivation for caring for a seriously ill family member at home, strength and stamina are needed. This is not new information. However, as our research shows, positive reinforcement is a minimally intrusive strategy that can have a greater impact on maintaining care at home when carers are under great strain.

## Availability of Data and Material (Data Transparency)

The datasets used and analyzed during the current study are available from the corresponding author on reasonable request.
